# A dynamic customized electronic health record rule based clinical decision support tool for standardized adult intensive care metrics

**DOI:** 10.1093/jamiaopen/ooae143

**Published:** 2024-12-11

**Authors:** Eric W Cucchi, Joseph Burzynski, Nicholas Marshall, Bruce Greenberg

**Affiliations:** University of Massachusetts Chan Medical School, Departments of Medicine, Worcester, MA 01655, United States; UMass Memorial Health, UMass Memorial Medical Center, Worcester, MA 01655, United States; University of Massachusetts Chan Medical School, Worcester, MA 01655, United States; University of Massachusetts Chan Medical School, Digital Health Program, Worcester, MA 01655, United States; University of Massachusetts Chan Medical School, Tan Chingfen Graduate School of Nursing, Worcester, MA 01655, United States; University of Massachusetts Chan Medical School, Worcester, MA 01655, United States; UMass Memorial Health, UMass Memorial Medical Center, Worcester, MA 01655, United States; University of Massachusetts Chan Medical School, Departments of Medicine, Worcester, MA 01655, United States; UMass Memorial Health, UMass Memorial Medical Center, Worcester, MA 01655, United States; University of Massachusetts Chan Medical School, Worcester, MA 01655, United States

**Keywords:** alert, alarm, critical care, patient monitoring, quality, safety, best practice advisories, clinical decision support tool, CDS, CDST, care gaps, our practice advisories

## Abstract

**Objectives:**

Many routine patient care items should be reviewed at least daily for intensive care unit (ICU) patients. These items are often incompletely performed, and dynamic clinical decision support tools (CDSTs) may improve attention to these daily items. We sought to evaluate the accuracy of institutionalized electronic health record (EHR) based custom dynamic CDST to support 22 ICU rounding quality metrics across 7 categories (hypoglycemia, venothromboembolism prophylaxis, stress ulcer prophylaxis, mechanical ventilation, sedation, nutrition, and catheter removal).

**Design:**

The dynamic CDST evaluates patient characteristics and patient orders, then identifies gaps between active interventions and conditions with recommendations of evidence based clinical practice guidelines across 22 areas of care for each patient. The results of the tool prompt clinicians to address any identified care gaps. We completed a confusion matrix to assess the sensitivity, specificity, accuracy, positive predictive value (PPV), and negative predictive value (NPV) of the dynamic CDST and the individual metrics.

**Setting:**

Tertiary academic medical center and community hospital ICUs.

**Subject:**

Customized Clinical Decision Support Tool.

**Measurements and Main Results:**

The metrics were evaluated 1421 times over 484 patients. The overall accuracy of the entire dynamic CDST is 0.979 with a sensitivity of 0.979, specificity of 0.978, PPV 0.969, and NPV 0.986.

**Conclusions:**

A customized, EHR based dynamic CDST can be highly accurate. Integrating a comprehensive dynamic CDST into existing workflows could improve attention and actions related to routine ICU quality metrics.

## Introduction

In 2003 Dr. Pronovost et al. published a seminal paper showing that setting daily goals improved care communication in the intensive care unit (ICU) and reduced length of stay.^1^ Since this time there has been a trend to establish daily multi-professional team-based discussions of standardized daily goals for each patient in the ICU.^2^^,^^3^ These daily discussions include both individualized (patient-specific) and standardized topics. The standardized topics are relevant to all critically ill patients. The exact topics can vary between institutions, but common core metrics involve glycemic control,^4–10^ sedation,^11^^,^^12^ venothromboembolism (VTE) prophylaxis,^13^ stress ulcer prophylaxis,^13–15^ tidal volume,^16^^,^^17^ nutrition,^18^ and decreasing hospital acquired infection risk including catheter associated urinary tract infections^19^ and central-line associated blood stream infections.^20^ The 2009 Society of Critical Care Medicine Guidelines include the recommendation that “process improvement is the backbone of achieving high quality ICU outcomes” and that “standardized protocols to facilitate measurable process and outcomes should be used and further developed in the ICU setting.”^2^ Despite these recommendations items are frequently missed.^3^^,^^21–24^

The office of the National Coordinator for Health Information Technology describes clinical decision support (CDS) as “providing individuals with knowledge and person-specific information, intelligently filtered, or presented at appropriate times, to enhance health and health care. CDS encompasses a variety of tools to enhance decision-making in the clinical workflow. These tools include computerized alerts and reminders to care providers and patients; clinical guidelines; condition-specific order sets; focused patient data reports and summaries; documentation templates; diagnostic support, and contextually relevant reference information, among other tools.”^25^ CDS have been shown to have a propensity to improve provider performance and improving process,^26^^,^^27^ despite a level of risk of biases in how the CDS were studied.^28^ Our dynamic CDST utilizes rule-based algorithms and integrates the output into the clinical workflows of bedside clinicians within the electronic health record (EHR). The output of the dynamic clinical decision support tool (CDST) allows clinicians to focus only when a gap between active conditions or interventions and evidence-based guidelines exist. Compared to a standard paper checklist, digital checklists may have better compliance with clinical standards.^21^ Our dynamic CDST looks to make the bedside clinician more efficient in identifying these gaps in care.

Currently, there is a dearth of reports in the medical literature regarding EHR embedded dynamic CDSTs in the ICU that focus on highlighting gaps in care. Much of the focus has been on disease specific tools, such as sepsis evaluation and management^29–32^ or acute kidney injury (AKI) identification,^33–35^ or pharmacy decision support.^36^ Conversely the ICU providers evaluate enormous quantities of patient specific data to safely make overly complex medical decisions. Our goal was to evaluate the accuracy of an EHR based tool to notify clinicians when standard quality metrics need to be acted upon a critically ill patient in the adult ICU.

## Materials and methods

Standardized evidence based clinical practice guidelines (CPGs) have been developed by multi-professional UMass Memorial Health (UMMH) clinical experts and approved by committee census and followed by all adult ICUs within the health system. Providers are encouraged to follow the approved CPGs but due to patient variability we know that occasionally these guidelines cannot be precisely followed. Two authors (E.C., B.G.) met with UMMH medical and nursing leaders and experts and based on these discussions a list of 22 separate metrics that are to be reviewed daily on each patient (eg, VTE prophylaxis, low tidal volume, the need for a urinary catheter or central venous catheter, etc.) were identified. All 22 of these metrics may not be relevant, or require action, for a particular patient on a particular day. For example, a patient may be not on anticoagulation for VTE prophylaxis due to increased bleeding risk, or a patient may still require a central venous catheter due to ongoing vasopressor requirements. As imperative as it is to review these standard metrics in each patient daily a static list of 22 metrics for each patient can be burdensome and monotonous due to possible irrelevance, or no action required secondary to obvious need, of a particular metric for a patient. In order to automate and dynamically alert clinicians as to which of the 22 metrics were relevant and require action on a specific patient each day we developed a dynamic CDST to support provider decision making regarding. The logic was designed based upon UMMH evidence-based CPGs associated with each metric. For example, the “Consider removal of central line” metric is based upon the recommendations of our Central Line Associated Blood Stream (CLABSI) CPG regarding prevention of CLABSIs and indications for placement and removal of central venous catheter. In addition to CPG guidance, each dynamic CDST was designed with expert input to ensure that the logic was applicable across multiple critical care subspecialties (medical, surgical, neuroscience, and cardiovascular). Due to inter-staff variability in documentation and information that lacks discrete data elements (eg, clinical reasoning that has been free texted) within the EMR we found that developing straightforward dynamic CDST rules based on discrete data elements was difficult. We had to account for the variability of data inputs by primarily using “sources of truth” in the EHR. As an example, we found that medication data from orders and MAR actions were far more reliable than provider note documentation. This allowed us to account for things such as inaccurate documentation or missed administrations of medications, when providers thought the patient was receiving a medication. Additionally, we had to develop dynamic CDST rules based upon the same discrete data elements the providers were evaluating to make their decisions. For example, the “Consider chemical VTE prophylaxis” metric alerts if the patient active mechanical VTE prophylaxis in place for at least 75% of the current day but does not have an order for an active chemical VTE prophylactic or a therapeutic dose anticoagulant (UFH, LMWH, DOAC, warfarin). If the patient is not coagulopathic (as defined by platelets <50, in last 3 days, INR ≥1.5 in last 3 days, aPTT >40 in last 3 days), does not have an active diagnosis of bleeding or hematoma, is not comfort care status, has not received >1 units of PRBCs in the prior 24 hours and is not ambulatory with mechanical prophylaxis the dynamic CDST will flag within the patient chart alerting the provider to an action that may prevent a care gap. In addition to documentation variability, we had to account of missing or absent data. This was handled in 1 of 3 ways. If the missing data was expected to be there and was absent (eg, missing chemoprophylaxis in a patient expected to have chemical VTE prophylaxis), it is treated as a missing and considered a negative finding. If the data is missing and is expected to be missing (eg, has a no central line when the patient does not require one), it is considered as a positive finding. Lastly, if a lab value is helpful for decision making and is absent (eg, an INR in determining the need for VTE prophylaxis) it is considered within the normal limits, making the assumption that the clinicians actively chose not to obtain a data point. Our binary alerts were displayed as a flag was an “X,” representing an actionable finding, whereas the absence of the “X” suggestion no action required. This flag was placed within columns within the patient census screen for each adult ICU unit represented that at least 1 criteria of the rule-based algorithm did not meet the expected guideline criteria.

Our goal was to validate the dynamic CDST against approximately 10% of the patient population. The patients were selected in real-time by the reviewers (J.B., E.C., and N.M.) based upon being actively admitted to an ICU bed on the days that analysis took place, which was at the convenience of the reviewer. The analysis took place between January 29, 2022, and August 30, 2022. Three researchers (J.B., E.C., and N.M.) manually reviewed patient charts in 1 of 9 adult ICUs. One researcher would review both patient data where dynamic CDST flagged a patient for a specific metric, and patient data where the dynamic CDST did not flag a specific metric for each patient reviewed. Each researcher would evaluate for the presence or absence of each data element required for a particular metric. As an example, for the “Consider chemical VTE prophylaxis” metric described above, each researcher would evaluate for the presence or absence of the documentation of compression boots or stockings, MAR actions of chemoprophylaxis administered or not administered, values of aPTT, INR and platelets compared to the algorithms cut off values, presence of a diagnosis that is associated with bleeding or hemorrhage, code status, transfusion records for blood administration, and activity orders. The purpose of this was to evaluate objectively if the criteria for an actionable flag was achieved or not.

Each metric within the dynamic CDST was considered a true positive, true negative, false positive, or false negative based upon its ability to identify an actionable flag to prevent a possible care gap (eg, missing VTE chemical prophylaxis). A true positive was consider a dynamic CDST flag that appropriately identified an actionable flag to prevent a possible potential gap in care, true negative was considered a dynamic CDST flag that appropriately did not fire when there was no actionable flag to prevent a possible gap in care, a false positive was considered a dynamic CDST flag that inappropriately identified an actionable flag to prevent a possible gap in care, and a false negative was considered a dynamic CDST flag that failed to fire when analysis of the patient’s chart indicated that it should have identified an actionable flag to prevent a possible gap in care.

The EHR logic was built within Epic version February 2022 (Verona, WI), primarily by 1 Epic physician builder (E.C.). Iterative testing was done on provisional dynamic CDST logic and adjustments made as performance gaps were identified.

The staffing, scheduling, governance structure, and models of critical care delivery were not changed during the study. The manuscript does not compromise intellectual property.

The dynamic CDSTs were evaluated against data from patient charts for any patient admitted to all but 1 of 9 UMMH adult ICUs. One community UMMH ICU was excluded because it did not use Epic at the time of the study. Since this study investigated the electronic model developed, the University of Massachusetts Chan Medical School Institutional Review Board felt it unnecessary to review this study because it did not meet the board’s guidelines or criteria for human subject research.

### Study population

UMMH, located in Worcester, MA, includes 4 community hospitals and the largest academic tertiary medical center in Central Massachusetts and, at the time of this analysis, utilizes Epic as the primary EHR. UMMH provides care to critically ill adults in 10 ICUs across 3 community hospitals and 1 academic medical center for a total of 127 ICU beds (24 mixed community ICU beds, 46 medical ICU beds, 16 neurotrauma ICU beds, 16 cardiothoracic and vascular ICU beds, and 25 surgical ICU beds).

### Statistical analysis

Using R version 4.2.1 (R: The R Project for Statistical Computing [r-project.org]) we calculated specificity, sensitivity, accuracy, positive predictive value (PPV) and negative predictive value (NPV) for each metric and the dynamic CDST overall. The PPV is the dynamic CDST correctly identified a gap in care and the NPV is the dynamic CDST correctly identified no gap in care. The dynamic CDST was evaluated a total of 1424 times against 484 patients’ data.

## Results

During the study period 4602 patients were admitted to the 9 adult ICUs. The study cohort of 484 comprised 10.5% of patients admitted during the study period. The study cohort was 43.6% female with an average age of 63, with a mean APACHE IV score of 70.68 (see Table 1).

**Table 1.** Baseline characteristics.

Characteristic	*n* (%)
Total cases	484
Mean age	63.05
Female	211 (43.6)
Male	273 (56.4)
Mean APACHE score	70.68
Mean APS	59.19
Median hospital LOS	14.33
Median unit LOS	8.11
Hospital deaths	101
ICU deaths	76
Surgical units	86 (17.77)
Medical units	180 (37.19)
Cardiovascular units	64 (13.22)
Neuroscience units	54 (11.16)
Community units	100 (20.66)

Abbreviations: APACHE, Acute Physiology and Chronic Health Evaluation; APS, Acute Physiology Score; LOS, Length of Stay.

The overall performance of the dynamic CDST showed a sensitivity of 97.9%, specificity of 97.9%, accuracy of 97.9%, PPV of 97%, and NPV of 98.6%. Each metric within the dynamic CDST was evaluated independently as well, see Table 2. Ten of the metrics within the dynamic CDST have an accuracy of 1 and “Needing Nutrition Started” had the lowest accuracy of 0.87.

**Table 2.** eICU population health clinical decision making tools model validation results.

*N*	Sensitivity	Specificity	Accuracy	PPV	NPV
Overall					
1421	0.979	0.978	0.979	0.969	0.986
Hypoglycemia (≤70 mg/dL)					
16	1	1	1	1	1
Consider chemical VTE prophylaxis					
33	1	0.966	0.97	0.8	1
Consider mechanical VTE prophylaxis					
22	1	1	1	1	1
Consider mechanical and/or chemical VTE prophylaxis					
33	1	0.962	0.97	0.875	1
Consider SUP					
18	1	1	1	1	1
Consider to discontinue SUP					
9	1	1	1	1	1
Low tidal volume (V_t_ ≤8 mLs/kg)					
37	1	1	1	1	1
Ready for sedation wean					
115	0.985	0.939	0.965	0.956	0.979
Ready for Spontaneous Breathing Trial					
16	1	1	1	1	1
Passed Spontaneous Breathing Trial: consider extubation					
39	1	1	1	1	1
Needs nutrition started					
46	0.714	0.938	0.87	0.833	0.882
Not meeting nutritional 80% of need					
53	1	0.973	0.981	0.941	1
Urinary catheter ready for removal					
130	1	0.944	0.985	0.979	1
Central line ready for removal					
149	0.944	0.974	0.96	0.971	0.949
Arterial line ready for removal					
189	0.972	0.983	0.979	0.972	0.983
PICC line ready for removal					
109	0.974	1	0.991	1	0.986
Pre-existing line of admit needs surveillance blood culture					
25	NaN	1	1	NaN	1
Rectal tube ready for removal					
66	1	1	1	1	1
Change Nasogastric tube to an Oralgastric Tube					
34	1	1	1	1	1
High risk Central Venous Catheter					
84	1	1	1	1	1
High risk arterial line					
58	1	0.968	0.983	0.964	1
High risk Hemodialysis line					
36	1	0.962	0.972	0.909	1
High risk PICC line					
67	1	0.967	0.97	0.778	1

Abbreviations: PICC, Peripherally Inserted Central Catheter; SUP, Stress Ulcer Prophylaxis; VTE, Venothromboembolism.

## Discussion

Standard quality topics should be evaluated daily for every patient to try to improve outcomes. There are a multitude of items to address, in areas such as glucose control, ventilator management, VTE and SUP prophylaxis, and infection control. Reviewing these items daily, for every patient, can be cumbersome and result in misses.^21^^,^^23^^,^^37^ These missed opportunities can result in poor outcomes. Having additional support can improve adherence to standard quality metrics.^21^^,^^24^^,^^37^ Improved adherence could lead to improved outcomes, such as decreasing line burden days.^24^ A dynamic CDST that is placed within a clinician’s workflow could be helpful to improving adherence if the clinician trusts the tool. The tool must be highly accurate to be useful and trusted. To be efficient it should prompt clinically relevant action and not simply documentation or attention where no change is warranted. The innovation in our dynamic CDST is that around the idea that it is dynamic. A static list of almost 2 dozen metrics that is required to be reviewed daily by a team on each patient is repetitive and can result in alarm fatigue, resulting in missed opportunities for action. The dynamic CDST discussed here, allows for clinicians to focus in on actionable items and sets the foundation to potentially decrease missed actionable opportunities which could lead to improved patient outcomes. For example, if central lines are more aggressively reviewed and removed daily, the overall line burden for a population could decrease, which could result in decreased CLABSI.

Dynamic CDSTs have been designed to assist busy clinicians in recognizing changes or conditions (Sepsis, AKI), and to attend at least one of the components we review here.^38^ Tools that aim to encourage early identification of sepsis have yielded varied results, which highlights the need for such decision support tools to be accurate.^39–42^ To our knowledge, this is the first report of a dynamic CDST that supports a comprehensive list of key topics of daily importance for critically ill patients. Our tool is also based on extensive, rule-based algorithms, and therefore holds the potential to decrease the workload of bedside clinicians by highlighting only the items that are likely to warrant a management change, or discussion of a change.

We have demonstrated that a custom built dynamic CDST tool based upon health system CPGs was highly accurate in both identifying patients whose care might be adjusted by specific actions, as well as identifying those with devices or standard support items who do not need adjustments to those items. By decreasing the number of items that need daily review by suppressing items that do not need attention, our dynamic CDST holds the promise of improving provider efficiency and overall quality of care if more focused attention results in improved compliance with standard recommendations or in decreased device burden.

Strengths of our study include careful manual detailed assessment of the dynamic CDST when it both fired and did not fire. We based our dynamic CDST rules on our CPGs, which could easily be replicable by other institutions. Both our CPGs and the topics our dynamic CDST assesses are based on commonly referenced literature. They do not differ significantly from similar guidance at other institutions, meaning the logic we designed can successfully be customized at other institutions. Our system uses a common, widely used electronic medical record system, and is detailed and thorough, increasing the likelihood that the prompts will be acted upon. Although we are a single health care system, we did evaluate this same dynamic CDST across 8 adult ICU populations including medical, surgical, trauma, transplant, cardiac, neuroscience, and community patients.

Limitations of our study include lack of a robust randomization process and that we are a single health care system, and our workflow might differ from those used at other institutions. It is possible that our practitioners are more consistent in documenting information in the recommended EHR location. This is essential for the dynamic CDST to find the data. While we showed that our customized, CPG concordant dynamic CDST is highly accurate, in this study we did not show that the tool led to changes in patient care. Our dynamic CDST, while thorough and detailed, was limited to the data the tool can access. Some useful information that if present would inform decision making (eg, if numerous attempts to place peripheral IVs have failed) is simply not available in the EHR.

The next step will be to study the clinical impact of the dynamic CDST. Will tools like our dynamic CDST have the impact that would be expected and result in improved adherence to clinical metrics? For example, does the dynamic CDST increase action in removing central lines and result in reduction in line days or catheter related blood stream infections? Evaluation of this type of dynamic CDST at other institutions would be helpful to understand its impact on other health systems and a larger population of patients. Using these types of tools could identify patient phenotypes (eg, based on complexity, chronic conditions, severity of illness, etc.) that have higher risks of care gaps or identify providers with high rates of nonadherence to metrics. Tools such as the one designed here could generate a reliable way for identifying care gaps at a Health System level. Now that discrete care gaps can be identified electronically, they could be tracked to help improve the care within a large population of patients within Health Systems. Additionally, these tools could be compared to the usefulness of disease-specific tools, and how they could be better integrated into provider workflows. Lastly, we are interested in qualitatively evaluating the affect our tool has upon the efficiency and satisfaction of the bedside provider compared to previous methods for addressing these standard met.

## Conclusion

A CPG supported rule based customized dynamic CDST within the EHR has remarkably high accuracy in providing actionable information to the bedside providers in real time. This tool holds promise to enhance patient care and the providers’ experience.

## Data Availability

The data underlying this article will be shared on reasonable request to the corresponding author.
